# Hyperglycaemia does not affect antigen-specific activation and cytolytic killing by CD8^+^ T cells *in vivo*

**DOI:** 10.1042/BSR20171079

**Published:** 2017-08-31

**Authors:** Asha Recino, Kerry Barkan, F. Susan Wong, Graham Ladds, Anne Cooke, Maja Wallberg

**Affiliations:** 1 Department of Pathology, University of Cambridge, Tennis Court Road, Cambridge CB2 1QP, United Kingdom; 2 Department of Pharmacology, University of Cambridge, Tennis Court Road, Cambridge CB2 1PD, United Kingdom; 3 Department of Biological Sciences, University of Warwick, Gibbet Hill Road, Coventry CV4 7AL, United Kingdom; 4 Diabetes Research Group, Institute of Molecular and Experimental Medicine, Cardiff School of Medicine, Cardiff University, Cardiff CF14 4XN, United Kingdom

**Keywords:** diabetes, hyperglycaemia, immunometabolism, mouse models, T-cells

## Abstract

Metabolism is of central importance for T cell survival and differentiation. It is well known that T cells cannot function in the absence of glucose, but it is less clear how they respond to excessive levels of glucose. In the present study, we investigated how increasing levels of glucose affect T-cell-mediated immune responses. We examined the effects of increased levels of glucose on CD8^+^ T-cell behaviour *in vitro* by assessing activation and cytokine production, as well as oxygen consumption rate (OCR), extracellular acidification rate (ECAR) and intracellular signalling. In addition, we assessed *in vivo* proliferation, cytokine production and cytolytic activity of cells in chemically induced diabetic C57BL/6 mice. Elevated levels of glucose in *in vitro* cultures had modest effects on proliferation and cytokine production, while *in vivo* hyperglycaemia had no effect on CD8^+^ T-cell proliferation, interferon γ (IFNγ) production or cytolytic killing.

## Introduction

Glucose is one of the important nutrients available to T cells, and is mostly taken up via Glut1 in these cells [1]. Glut1 is up-regulated upon activation, which leads to increased glucose uptake and glycolysis to promote growth, proliferation, cell survival and differentiation [2]. As a result of this, Glut1 deficiency in T cells decreases effector cell expansion and the ability to induce inflammatory disease *in vivo* [1]. Recent studies have clarified how T cells up-regulate their anaerobic glycolysis during a rapid effector response, and how this type of rapid but low efficiency generation of energy must be replaced by engagement of the mitochondria and fatty acid oxidation [3] or the ability to sustain high levels of ATP generation through elevated glycolysis [4] for the cells to differentiate into long-lived memory T cells. In contrast, Foxp3^+^ Treg favours fatty acid oxidation [5,6], and induction of anergy in effector T cells reduces their metabolism [7]. The metabolism of T cells is a drugable target, and indeed the mammalian target of rapamycin (mTOR) is at the centre of the cell response to nutrient availability and dictates cell decisions to grow and differentiate [8–10].

We were interested in how an abundance of glucose, as is the case in diabetes, affects the adaptive immune system. As competition for resources can lead to suppression of immune responses [11], while the elevated presence of glucose has been reported to both boost the immune responses to tumours [11] and enhance the survival of mice after administration of lethal doses of influenza virus [12], it seemed likely that elevated levels of glucose could enhance immune responses. In order to provide sufficient levels of glucose, many cell culture media contain ‘diabetic’ levels of glucose, with concentrations often in the 12–15 mM range or even higher, which is well above the levels seen in healthy people (below 6 mM in the fasting state and below 7.8 mM 2 h postprandial). On the other hand, patients with diabetes have numerous and more serious infections than the healthy control subjects [13,14], and decreased responses to vaccination [15,16] indicating that elevated glucose levels do not boost immune responses *in vivo*.

Here, we investigated how increasing levels of glucose *in vitro*, varying from a low but physiologically normoglycaemic concentration of 5.5 mM (1 g/l) up to an emphatically hyperglycaemic environment of 25 mM (4.5 g/l), affected T-cell behaviour. We have also investigated the *in vivo* effects of hyperglycaemia (ranging between 15 and 25 mM), on OVA-specific CD8^+^ T-cell proliferation, cytokine production and cytolytic killing in streptozotocin (STZ)-induced diabetic C57BL/6 mice.

## Methods

### Mice

OT-I were bred at the University of Cambridge and maintained under specific pathogen-free conditions. Male C57BL/6 mice (Charles River) were used between 6 and 10 weeks of age. Mice were housed in IVC with free access to standard chow and water. The present study was carried out in accordance with U.K. Home Office Regulations (project licence number 80/2442 and 70/8442).

### STZ-induced diabetes

Male C57BL/6 mice were given STZ (Sigma, 40 µg/g body weight) dissolved in citrate buffer (pH 4.5) intraperitoneally for 5 days. Diabetes normally developed within 10–14 days with no signs of STZ-induced lymphopaenia (Supplementary Figure S1). Glycosuria was detected using Diastix strips (Bayer Diagnostics) and diabetes confirmed by a blood glucose measurement of >13.3 mM, using a Breeze2 blood glucose meter (Bayer).

### Antibodies and flow cytometry

Cells were resuspended in FACS buffer (PBS + 0.5% BSA) filtered through 30-µm CellTrics filters (Partec), incubated with Fc block (eBioscience), stained with antibody, washed and resuspended in PBS. 7AAD (BD Bioscience) was used to assess cell death. Data were collected on a Cyan Cytometer (DAKO) and analysed using FlowJo (TreeStar Inc.). For intracellular cytokine staining, the cells were stimulated with PMA (50 ng/ml) and ionomycin (2000 ng/ml) for 5 h. Brefeldin A (5 µg/ml) was added for the last 3 h. After surface marker staining, the cells were washed, fixed, permeabilized (intracellular staining kit, eBioscience), and stained for detection of cytokine.

### T-cell activation for functional assays

Cells were isolated from spleen and lymph nodes and cultured in low glucose (5.5 mM) DMEM with 10% FBS, 1% penicillin-streptomycin, and β-mercaptoethanol supplemented with additional glucose as indicated. Lymphocytes (2 × 10^5^) were stimulated as appropriate (see below) for 3 days in the presence of the indicated glucose concentrations at 37°C with 5% CO_2_. OT-I cells were stimulated either with the OVA peptide SIINFEKL or the lower affinity altered peptide ligand SIIGFEKL (both from Sigma) as indicated. Proliferation was assessed by CFSE staining (5 µM). After gating on CD8^+^ T cells, the percentage of proliferating cells in each population was determined. Supernatant cytokine analysis was performed with cytometric bead array (eBioscience) as recently described [17], and ATP content in cultures was assessed using the CellTiter-Glo® Luminescent Cell Viability Assay (Promega) in accordance with the manufacturers’ instructions. The cells were cultured in 96-well plates at a concentration of 2.5 × 10^4^ cells per well in 100 µl of the indicated culture medium. For analysis, the supernatants were transferred into a 384-well Optiplate (PerkinElmer) and luminescence read using a Mithras LB 940 (Berthold Technology).

### Measurements of T-cell metabolism

Naïve OT-I CD8^+^ T cells were isolated using MACS beads (Miltenyi) according to the manufacturer’s instructions. For studies of activated cells, OT-I splenocytes were cultured for 5 days in the presence of 10 ng/ml SIINFEKL peptide and 10 U/ml IL-2 (PeproTech). Naïve cells were seeded in a 96-well seahorse plate at 3 × 10^5^ cells per well, and activated cells were seeded at 1.5 × 10^5^ cells per well, and analysed using the Mitostress kit (Agilent Technologies) according to the manufacturers’ instructions. Seahorse assay medium (Agilent Technologies) was supplemented with the indicated glucose concentration, 1 mM glutamine and 1 mM pyruvate. Oligomycin was administered at 1.5 µM, FCCP at 1 µM and rotenone/antimycin A at 1 µM (all from Agilent Technologies). Oxygen consumption rate (OCR) and extracellular acidification rate (ECAR) were measured using a XF96 Seahorse analyser. ATP turnover was derived from the difference in OCRs between basal respiration and inhibition after oligomycin administration according to the manufacturers’ instructions.

### Ca^2+^ flux assay

Single cell suspensions (2 × 10^6^/ml) were incubated with Indo-1 (3 µM) for 30 min at 37°C. The cells were then washed twice in HBSS (Sigma), and resuspended in HBSS + 0.2% FBS at 10^6^/ml and aliquoted into FACS tubes, 1 ml per tube. Baseline activity was measured for 1 min, and then stimulating antibodies (anti-CD3, clone 145-2C11, 2 µg/ml and anti-CD28, clone 37.51, 10 µg/ml) were added for another 7 min of recording. MFI for Indo-1 was plotted for each minute of stimulation.

### Zap phosphorylation assay

CD8^+^ T cells were sorted using MACS (Miltenyi), seeded in V-bottom plates (2 × 10^5^/well) and incubated with stimulating antibodies (anti-CD3, clone 145-2C11, 2 µg/ml and anti-CD28, clone 37.51, 10 µg/ml) for the indicated time with the indicated concentration of glucose at 37°C. After stimulation, the cells were immediately fixed in 4% PFA for 30 min, then washed in PBS and stored in ice-cold methanol at –20°C, stained with anti p-Zap319 and detected with anti-rabbit IgG Fab2 Alexa 647 (Molecular Probes).

### *In vivo* proliferation assays

For C57BL/6 mice, OVA was emulsified in Incomplete Freund’s Adjuvant (IFA) at 25 µg per dose and injected subcutaneously (sc) into the left haunch. Eight days later, 1 × 10^6^ CFSE-labelled OT-I cells were transferred intravenously (iv) into the indicated recipient. Seventy-two hours later the draining inguinal lymph node and the control non-draining lymph node were harvested and proliferation was assessed through analysis of dilution of CFSE signal in CD8^+^7AAD^−^B220^−^ cells.

### *In vivo* CTL assay

Male C57BL/6 mice were immunized with SIINFEKL peptide at 25 µg/dose emulsified in IFA (Sigma) sc in the left haunch. Ten days later, targets were injected. Syngeneic splenocytes were either peptide-pulsed (100 nM, 30 min, 37°C) and subsequently labelled with 10 µM CFSE, or non-pulsed and labelled with 1 µM CFSE. The splenocyte populations were then mixed at 50:50, and 10^7^ cells were injected in the tail vein. Twenty-four hours later, the inoculum draining and control side inguinal lymph nodes were collected, and the ratio of CFSE^hi^ compared with CFSE^intermediate^ cells compared with non-immunized controls to calculate % of specific killing of peptide-pulsed targets.

### Statistical analysis

Differences between groups were tested using the Student’s *t* test, significant *P*-values are indicated with *(*P*≤0.05), **(*P*≤0.01), ***(*P*≤0.001) or ****(*P*≤0.0001). Comparison of multiple groups in the Seahorse assays was performed using two-way ANOVA followed by Dunnett’s multiple comparison test. All data analyses were performed using GraphPad Prism 7 software.

## Results

### OT-I cell proliferation to high affinity, but not low-affinity peptide, is increased when glucose levels are raised

**Figure 1:**
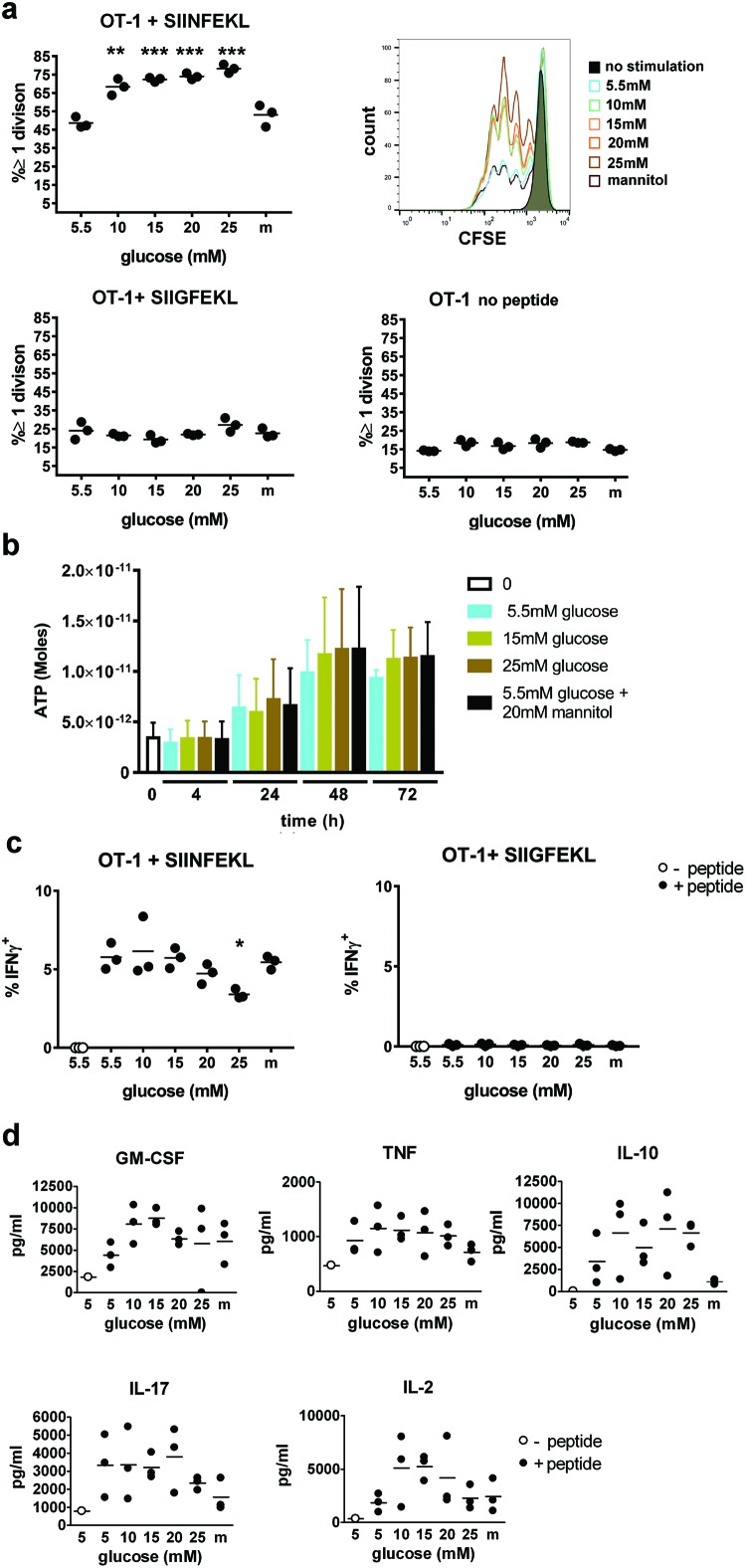
Effects of increasing levels of glucose in culture medium on OT-I cell proliferation and cytokine production (**a**) Proliferation was assessed by CFSE dilution in OT-I cells in response to SIINFEKL (left panel), the low-affinity altered peptide SIIGFEKL (middle panel) and no peptide control (right panel). (**b**) ATP content at different time points in response to stimulation with anti-CD3 and anti-CD28 antibody in different concentrations of glucose or mannitol control. (**c**) IFNγ production was assessed using intracellular staining in cultures with SIINFEKL peptide (left) and low affinity altered peptide (right). (**d**) Cytokine production in OT-I cultures in response to SIINFEKL peptide in the presence of increasing concentrations of glucose or 25 mM mannitol (m) as an osmolarity control was assessed using cytokine bead array. The results are representative of at least three experiments. Differences between groups were tested using the Student’s *t*test, significant *P*-values are indicated with *(*P*≤0.05), **(*P*≤0.01), ***(*P*≤0.001) or ****(*P*≤0.0001). If no *P*-value is indicated, there was no significant difference between the groups.

We assessed the *in vitro* proliferation of OT-I cells, which are CD8^+^ T cells reactive to ovalbumin peptide 257–264 (SIINFEKL) presented on C57BL/6 MHC class I molecule H2K^b^. Increasing levels of glucose resulted in increased proliferation of these cells in response to their cognate peptide (Figure 1a, top left panel, with representative CFSE traces in the top right panel). However, this proliferative change with increasing levels of glucose were not seen with the low-affinity peptide ligand SIIGFEKL (Figure 1a, bottom left panel) or medium alone (Figure 1a, bottom right panel), indicating that increased glucose did not alter the threshold for activation. Cells cultured in an excess of culture medium did not grow more in higher concentrations of glucose, as reflected in the ATP content in cultures at different time points (Figure 1b). In contrast with increased proliferation seen in high glucose cultures, we saw a decrease in interferon γ (IFNγ) production in cultures with glucose levels of 25 mM (Figure 1c, left panel), and no IFNγ produced in response to the altered peptide ligand at any glucose concentration (Figure 1c, right panel). Production of GM-CSF, TNF, IL-10, IL-17 and IL-2 appeared unaffected, with a trend towards increased production at moderate levels of glucose (10–15 mM) and a decrease at high levels (20–25 mM) (Figure 1d). To control the changes in osmolarity caused by increased glucose concentrations, we included 20 mM mannitol, a sugar with similar molecular weight to glucose but not metabolized by cells, added to a 5.5-mM glucose base medium.

### Elevated levels of glucose do not alter OCR in naïve or activated OT-I cells

**Figure 2:**
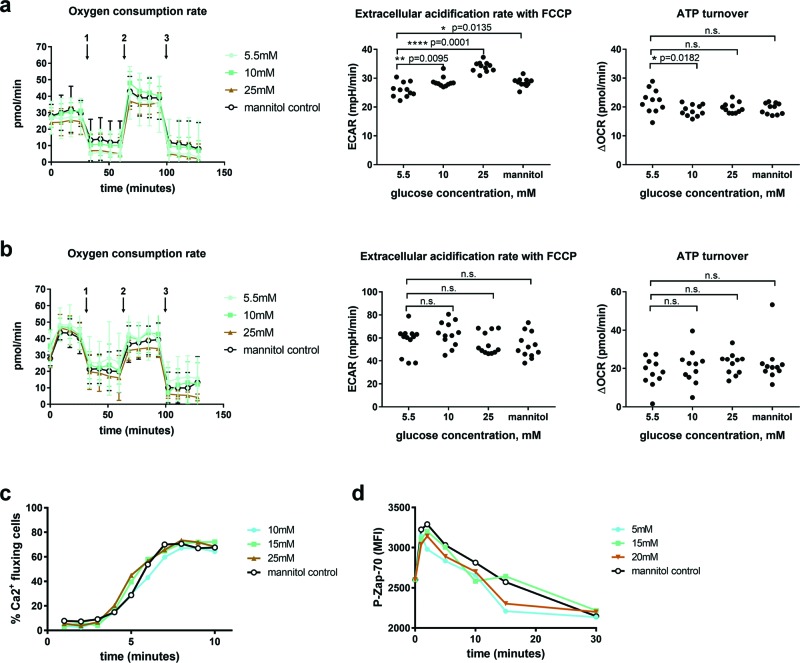
Effects of increasing levels of glucose on OT-I cell metabolic activity and intracellular signalling (**a**) OCR was determined for naïve OT-I cells in response to compounds that target different parts of the mitochondrial electron transport chain ((a), left panel). Oligomycin, FCCP and rotenone/antimycin A were administered at the indicated time points (indicated in the figure with arrows numbered 1, 2 and 3 respectively) followed by four separate measurements for each condition. The ECAR was determined after addition of FCCP for maximum activation ((a), middle panel). Each data point indicate the average of the four measurements in one well. Eleven replicates per condition were assessed. ATP turnover was calculated from the difference in OCR between the basal and oligomycin stimulated conditions ((a), right panel). (**b**) OCR was determined for activated OT-I cells as described above ((b), left panel), as was ECAR ((b), middle panel) and ATP turnover ((b), right panel). (**c**) Ca^2+^ fluxing and (**d**) Zap-70 phosphorylation were determined in cells after activation in culture media with different concentrations of glucose using flow cytometry. The data are representative of at least two experiments. Differences between groups were determined through two-way ANOVA followed by Dunnett’s multiple comparison test with *P*-values below 0.05 considered significant.

To assess whether increased glucose concentration changed the metabolic activity of the OT-I cells, we assessed their OCRs (Figure 2a,b, left panels) and ECAR (Figure 2a,b, middle panels) in response to drugs that affect the electron transport chain [3]. Oligomycin inhibits the ATP synthase stopping mitochondrial ATP generation, FCCP is a protonophore which uncouples ATP synthesis from the electron transport chain by letting H^+^ ions into the matrix independent of the ATP synthase while rotenone/antimycin A inhibit the complex I and III respectively, leading to complete shut down of the electron transport chain. We found that increasing the levels of glucose modestly increases the ECAR of naïve cells in a dose-dependent manner (Figure 2a, middle panel) but does not affect the already higher ECAR of activated cells (Figure 2b, middle panel). The ATP turnover, determined by the difference in OCR between basal levels and the levels after oligomycin inhibition of the ATP synthase, were unaffected by glucose concentration in both naïve cells (Figure 2a, right panel) and activated cells (Figure 2b, right panel). Furthermore, the immediate activation of T cells as determined through Ca^2+^ fluxing (Figure 2c) and Zap-70 phosphorylation (Figure 2d) was also unaffected by glucose concentration.

### Hyperglycaemia does not affect OVA-specific proliferation, IFNγ production or CTL killing *in vivo*

**Figure 3:**
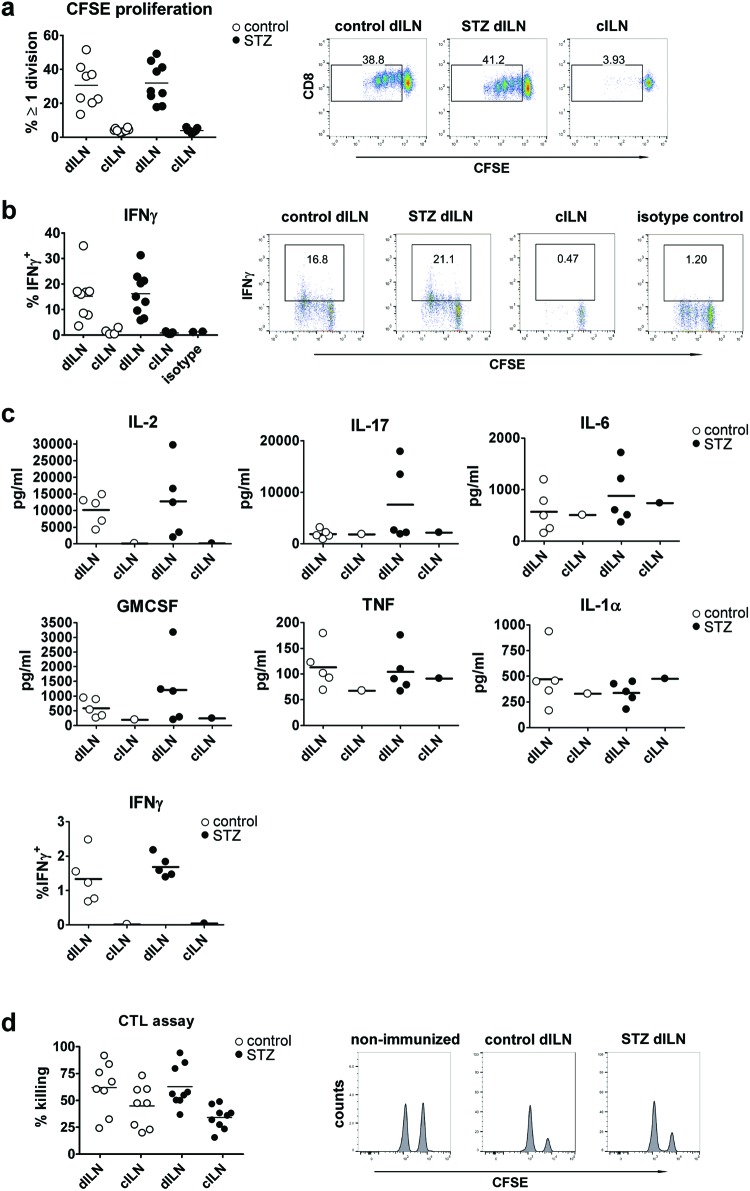
Hyperglycaemia does not affect immune responses *in vivo* in STZ-induced diabetic C57BL/6 mice (**a**) *In vivo* proliferation of CFSE-labelled OT-I cells was assessed in response to an inoculum containing OVA in control mice compared with mice rendered diabetic through injection of STZ. The left panel shows the result of one experiment with each dot representing one mouse, assessing either the inoculum-draining lymph node (dILN) or the control inguinal lymph node (cILN). The right panels show representative FACS plots. (**b**) IFNγ production in cells from the experiment described in (a), stimulated *ex vivo* with PMA for 4 h. The left panel shows the result of one experiment with each dot representing one mouse, the right panels show representative FACS plots. (**c**) Cytokine production in lymphocytes from mice immunized with OVA as above and restimulated *in vitro* with SIINFEKL peptide. (**d**) *In vivo* killing assay comparing the antigen-specific killing of SIINFEKL-pulsed syngeneic splenocytes during a 24-h period in mice immunized with the same peptide 8 days previously. The left panel shows the result of one experiment with each dot representing one mouse; the right panels show representative FACS plots. The results are representative of at least three experiments, and differences between groups were tested using the Student’s *t*test.

To assess whether any of the modest differences recorded *in vitro* were of importance *in vivo*, we performed experiments in C57BL/6 mice, which had been rendered diabetic using low-dose STZ injection. Control or hyperglycaemic C57BL/6 mice were immunized with OVA in the left haunch, and the proliferation of injected CFSE-labelled OT-I cells was assessed in the inoculum-draining left inguinal lymph node and the control right inguinal lymph node. There was no difference in how well the transferred OT-I cells proliferated in the diabetic hosts compared with control hosts (Figure 3a). To further assess the properties of the activated OT-I cells, the cells from the lymph nodes were restimulated briefly *in vitro* with PMA and ionomycin, and IFNγ production was recorded. There was no difference between the groups (Figure 3b). The inoculum-draining and non-draining lymph nodes from immunized mice were also restimulated with SIINFEKL peptide for measurement of production of other cytokines. There was no difference in the production of IL-2, IL-17, IFNγ, GM-CSF, TNF, IL-6 or IL-1α (Figure 3c). We also plotted the levels of cytokine and proliferation against measured blood glucose level at the end of the experiment, but found no correlation in any experiment (results not included). *In vivo* cytolytic T lymphocyte (CTL) assays demonstrated no difference in the capacity for OVA-specific CTL cytotoxicity in diabetic hosts compared with controls (Figure 3d).

## Discussion

We have investigated how levels of glucose in the diabetic range affect T-cell responses both *in vitro* and *in vivo*. We find that hyperglycaemia has modest effects on proliferation and cytokine production *in vitro*, which could simply reflect the fact that an *in vitro* culture has to adapt to the amount of nutrient available in the well*.* When the cells are cultured in excess volumes of media, as in the cultures prepared to assess ATP content, no difference in the accumulation of ATP could be detected in cultures with higher levels of glucose. To support this, we find that OCR and ATP turnover of both naïve and activated OT-I cells are unaffected by the hyperglycaemic conditions, and that initial intracellular activation events after T-cell receptor (TCR) ligation are unaltered by hyperglycaemia. Interestingly, we find that naïve OT-I cells demonstrate increased ECAR in hyperglycaemic conditions, and it remains to be determined if this has any biological significance. *In vivo* CD8^+^ T-cell proliferation and cytokine production was unaffected in diabetic C57BL/6, as was *in vivo* cytolytic killing. This finding is in contrast with a previous study, which demonstrated greater survival of tumour cells in STZ-induced diabetic mice [18]. It is however possible that the elevated glucose levels in diabetic mice affect not only CTL but also the tumour cells, and this may contribute to their greater survival. An important point to make here is the difference between STZ protocols. Many groups administer one high dose of 200 µg/g body weight [18–20] and may see a resulting down-regulation of immune responses. STZ is a glucosamine–nitrosourea that causes DNA damage, and is particularly toxic to β-cells as it is taken up via the Glut2 transporter, which is expressed in β-cells and to a lower extent in kidney, liver and small intestine. However, at high doses STZ can be toxic to other cell types as well, which is demonstrated by the lymphopenia seen in high-dose treated mice [19]. The injection protocol used in our study uses repeated low dose injections of 40 µg/g body weight, which avoids off target effects, and no lymphopenia was recorded as shown in the Supplementary Data (S1).

Health complications such as changes in immune reactivity in diabetes are caused by a complex network of interacting mechanisms, and it is difficult to determine which effects are caused by excess glucose itself, and how that effect is exerted. Hyperglycaemia has effects on the innate immune system in that it can inhibit neutrophil migration, phagocytosis, superoxide production and microbial killing [21,22] and decrease the production of antimicrobial peptides [23]. Neutrophils have been reported to take up less antigen in a hyperglycaemic host [24], which could indirectly lead to depressed T-cell responses, as they may not receive optimal antigen presentation. Hyperglycaemia also affects the ability of tolerogenic DC to induce generation of antigen-specific tolerance in T cells [25], and there are reports that hyperglycaemia can induce expression of proinflammatory cytokines like IL-17 in CD4^+^ T cells [26,27]. All these effects on immune cells may contribute to altered immune status in diabetic patients.

In the present study, we demonstrate that antigen-specific proliferation and killing by OT-I cells are unaffected by hyperglycaemia *in vivo*, indicating that an abundance of glucose does not in itself either suppress or boost short-term T-cell responses. It remains to be determined whether long-term effects of hyperglycaemia may alter antigen presentation to T cells, or the maintenance of the T cells themselves, thus affecting the formation and maintenance of T-cell memory.

## Supporting information

**supplementary Figure 1:** S1. Absolute numbers of cells in secondary lymphoid tissue in male C57BL/6 mice 2 weeks after repeated low dose streptozotocin administration.

## Abbreviations

CTL: cytolytic T lymphocyte
ECAR: extracellular acidification rate
IFA: incomplete Freund’s adjuvant
IFNγ: interferon γ
OCR: oxygen consumption rate
sc: subcutaneously
STZ: streptozotocin
OVA: ovalbumin
IL: interleukin
MFI: mean fluorescence intensity
