# Supplementary material for: Hyperglycaemia does not affect antigen-specific activation and cytolytic killing by CD8+ T cells in vivo
Source: Biosci Rep. 2017 Aug 31;37(4):BSR20171079. doi: 10.1042/BSR20171079 (PMC5634402; doi:10.1042/BSR20171079)
Supplement: Supplementary file 1 [file bsr20171079_Supp1.pdf]

# Supplementary figure 1

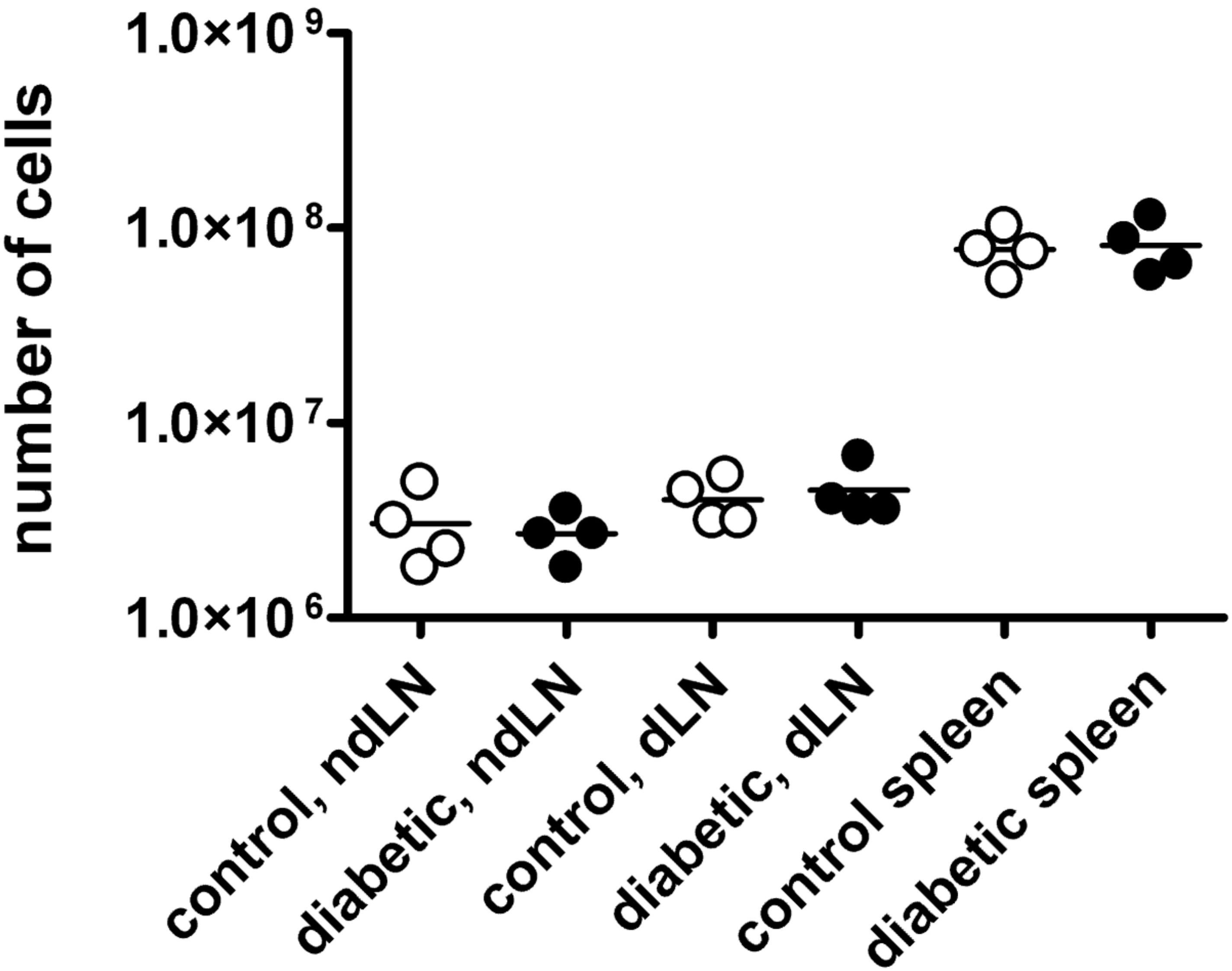


S1. Absolute numbers of cells in secondary lymphoid tissue in male C57BL/6 mice 2 weeks after repeated low dose streptozotocin administration.
